# Plasticity in Neuroblastoma Cell Identity Defines a Noradrenergic-to-Mesenchymal Transition (NMT)

**DOI:** 10.3390/cancers13122904

**Published:** 2021-06-10

**Authors:** Margot Gautier, Cécile Thirant, Olivier Delattre, Isabelle Janoueix-Lerosey

**Affiliations:** 1 Institut Curie, PSL Research University, Inserm U830, Equipe Labellisée Ligue Contre le Cancer, 75005 Paris, France; margot.gautier@curie.fr (M.G.); cecile.thirant@curie.fr (C.T.); Olivier.Delattre@curie.fr (O.D.); 2 SIREDO: Care, Innovation and Research for Children, Adolescents and Young Adults with Cancer, Institut Curie, 75005 Paris, France

**Keywords:** neuroblastoma, transdifferentiation, identity, noradrenergic, mesenchymal, transcriptomic profile, epigenetic state, EMT

## Abstract

**Simple Summary:**

Neuroblastoma arises in the developing sympathetic nervous system and represents the most common extracranial pediatric solid tumor. High-risk patients often experience relapse despite intensive multimodal therapies. Consistent with previous reports describing various phenotypes in neuroblastoma cell lines, recent studies precisely characterized two distinct cell identities based on transcriptomic and epigenetic profiles. In this review, we focus on the description of these two cell states, which we define as noradrenergic (NOR) and mesenchymal (MES), and discuss the plasticity between them. The differences in chemoresistance and invasion/migration properties of these two identities may have important clinical relevance. Deciphering the mechanisms of transdifferentiation from a NOR to a MES identity, which is reminiscent of the well-known epithelial-to-mesenchymal transition, is a key step to better understand neuroblastoma biology and improve therapeutic management of patients.

**Abstract:**

Neuroblastoma, a pediatric cancer of the peripheral sympathetic nervous system, is characterized by an important clinical heterogeneity, and high-risk tumors are associated with a poor overall survival. Neuroblastoma cells may present with diverse morphological and biochemical properties in vitro, and seminal observations suggested that interconversion between two phenotypes called N-type and S-type may occur. In 2017, two main studies provided novel insights into these subtypes through the characterization of the transcriptomic and epigenetic landscapes of a panel of neuroblastoma cell lines. In this review, we focus on the available data that define neuroblastoma cell identity and propose to use the term noradrenergic (NOR) and mesenchymal (MES) to refer to these identities. We also address the question of transdifferentiation between both states and suggest that the plasticity between the NOR identity and the MES identity defines a noradrenergic-to-mesenchymal transition, reminiscent of but different from the well-established epithelial-to-mesenchymal transition.

## 1. Introduction

Neuroblastoma is the most common extra-cranial malignant solid tumor of childhood. These tumors develop from the peripheral sympathetic nervous system that derives from multipotent neural crest cells (NCCs) [1,2,3]. Neuroblastoma accounts for 8% to 10% of pediatric cancers [4,5,6] with a median age at diagnosis of 18 months [7]. Approximately 40% of the patients are younger than 1 year at diagnosis [7]. Neuroblastoma is characterized by its heterogeneity of clinical presentation. Tumors are observed at different localizations: they arise most commonly in the adrenal gland and in the abdomen, but they can develop also from any site along the sympathetic ganglionic chain from the neck to the pelvis [8]. At diagnosis, tumors can be either localized or can be disseminated with metastasis mostly in bones, bone marrow, liver, or skin [8,9]. Heterogeneity also concerns the different possible outcomes, ranging from spontaneous regression without any medical intervention or differentiation into a localized benign ganglioneuroma [10,11,12] to malignant progression with metastatic spread and poor patient survival, despite intensive treatments. Neuroblastoma is composed of small round cells called neuroblasts. According to the International Neuroblastoma Pathology Classification (INPC), neuroblastic tumors are classified as stroma-rich or stroma-poor according to the presence and proportion of Schwann cells [13]. Stroma-poor neuroblastomas are stratified between undifferentiated, poorly differentiated, and differentiating tumors according to the differentiation grade of tumor cells.

More than 4 decades ago, it was reported that neuroblastoma cells may present with diverse morphological and biochemical properties in vitro [14]. This publication described and defined two types of phenotype called N-type (neuroblast) and S-type (substrate-adherent) [14]. The ability of neuroblastoma cells to interconvert bidirectionally in vitro from an N-type to an S-type state was then described in several patient-derived cell lines. Whereas a morphological and biochemical intermediate I-type between N- and S-types has been characterized [15], the mechanisms underlying cell interconversion, also called transdifferentiation, remained largely unknown.

This review focuses on the various phenotypes and properties of neuroblastoma cells and the related plasticity between different states. In the very recent years, the characterization of the transcriptomic and epigenetic landscapes associated with the distinct phenotypes of neuroblastoma cells has revealed that epigenetic regulation shapes cell identity. Core regulatory circuitries (CRCs) of specific transcription factors (TFs) controlling the gene expression program of neuroblastoma have been characterized for the two previously described N- and S-type states, further defined as noradrenergic and mesenchymal states. The differences in chemoresistance and invasion/migration properties between the two cell types and their possible interconversion may have important clinical relevance. Several studies have reported that some drug treatments may induce mesenchymal features in neuroblastoma cells in vitro. These studies often referred to an epithelial-to-mesenchymal transition (EMT) in neuroblastoma. We propose here that such plasticity rather defines a noradrenergic-to-mesenchymal transition (NMT).

## 2. Various Phenotypes and Biochemical Properties of Neuroblastoma Cells

### 2.1. Heterogeneity of Neuroblastoma Cell Phenotype: N- and S-Types

**Figure 1:**
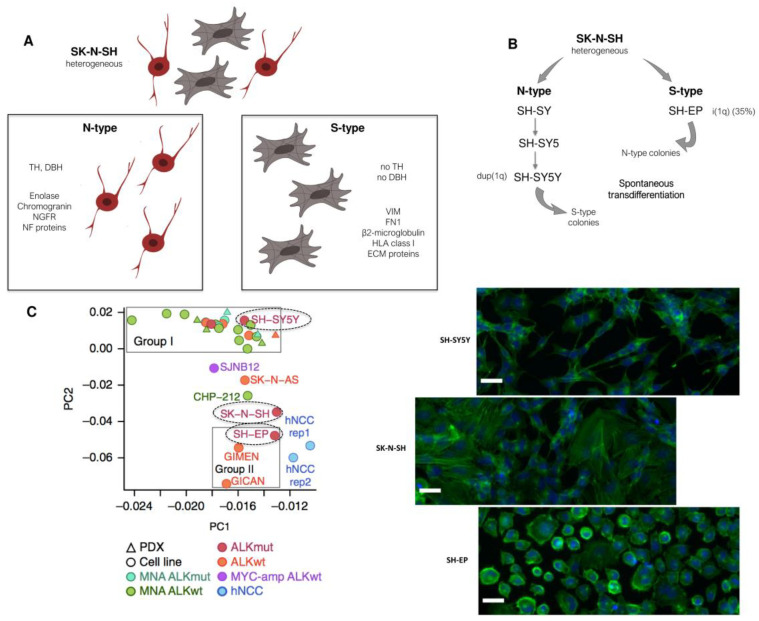
Heterogeneity of cell phenotype and identity in neuroblastoma cell lines. (**A**) The SK-N-SH cell line is heterogeneous with two distinct populations. The N-type population is constituted by cells with neurites, expressing the noradrenergic enzymes tyrosine hydroxylase (TH) and dopamine-β-hydroxylase (DBH) and markers such as enolase, chromogranins, neurofilaments (NF) proteins, or the NGF receptor. The S-type population includes substrate-adherent cells expressing markers including vimentin (VIM), fibronectin (FN1), β2-microglobulin, extracellular matrix (ECM) proteins, or HLA class 1 antigens. (**B**) Sub-lines derived from the heterogeneous SK-N-SH. The thrice-cloned SH-SY5Y sub-line presents an N-phenotype with a karyotype with a duplication of the chromosome 1q (dup(1q)), whereas the SH-EP sub-line presents an S-phenotype with an isochromosome 1q (i(1q)) in 35% of the cells. Interestingly, both cell types can undergo a spontaneous bidirectional interconversion. (**C**) Analysis of the SE landscape of the SK-N-SH, SH-SY5Y, and SH-EP cell lines revealed that epigenetic regulation shapes cellular identity (principal component analysis based on super-enhancer scores. Image is from “Heterogeneity of neuroblastoma cell identity defined by transcriptional circuitries” by Boeva, V. et al., 2017, Nature Genetics, 49, 1408–1413, Reprinted with permission from Ref. [19]. Copyright 2021 Springer Nature). The SH-SY5Y cell line is included in group I corresponding to a noradrenergic identity, while the SH-EP cell line is in group II, close to human NCC lines. The heterogeneous SK-N-SH cell line is included in an intermediate group. The different phenotypes of the cells of the corresponding cell lines are shown by immunofluorescence with Phalloïdin (green) and DAPI (blue). Scale bar = 20 µm.

The SK-N-SH cell line is one of the first neuroblastoma cell lines that has been shown to exhibit two highly different phenotypes [14]. This cell line was derived from the bone marrow of a four-year-old girl with neuroblastoma treated with surgery, radiotherapy, and chemotherapy. The SK-N-SH cell line comprises two populations with different morphologies called N- and S- types (Figure 1A). The N-type population is neuroblast-like with small spiny and dense cells with scant cytoplasm. These cells have delicate neuritic processes, attach poorly to the substrate and grow as focal aggregates. The S-type corresponds to large flattened cells that attach rapidly and strongly to the substrate [14]. Importantly, the N-type population is characterized by activities for neurotransmitter enzymes such as dopamine-β-hydroxylase (DBH) and tyrosine hydroxylase (TH), while none of those catecholamine activities are detected in the S-type population. In contrast, S-type cells were described to exhibit tyrosinase activity [16]. Further characterization of N- and S-type was performed using several extracellular matrix (ECM) proteins and intermediate filaments, known to be cell- and tissue-specific. Whereas N-type cells expressed NF68 (*NEFL*), NF150 (*NEFM*), and NF200 (*NEFH*), S-type cells produced the intermediate filament vimentin (*VIM*) and fibronectin (*FN1*), responsible for flatness and adhesiveness [15]. Previous studies have shown that S-type cells produce large amounts of stromal collagens of isotypes I and III (*COL1A1* and *COL3A1*) [17,18].

### 2.2. Interconversion between the N- and S-Types Relies on Two Distinct Differentiation Programs

The heterogeneous SK-N-SH cell line was sub-cloned into three successive N-type sub-lines, called SH-SY, SH-SY5, and SH-SY5Y, and into an S-type sub-line called SH-EP (Figure 1B) [20]. The morphological properties of these cell lines resumed the two populations of the parental SK-N-SH cell line. Indeed, SH-SY cells were neuroblast-like in appearance and expressed TH and DBH activities, whereas SH-EP cells were flattened, did not have neuritic processes, and did not express activity for any of the two enzymes. Interestingly, without any treatment, the thrice-cloned SH-SY5Y sub-line exhibited 0.3% of S-type colonies after 20 weeks in culture. Conversely, three out of nine subclones that grew from the SH-EP cell line had an N-phenotype after 10 to 12 weeks in culture. The authors excluded a potential contamination by showing that the isochromosome 1q, present in 35% of the SH-EP cells and absent in the SH-SY5Y cell line, was present in these N-type colonies derived from the SH-EP culture. Moreover, these colonies that changed into an N-type morphology expressed DBH activity showing that these cells also acquired biochemical properties of neuroblastic cells. These results showed for the first time that both cell types can undergo a spontaneous bidirectional interconversion with coordination in the biochemistry of these cells [20] (Figure 1B).

In a further step, molecular markers including cell surface differentiation antigens were searched for to confirm the neuronal phenotype of N-type cells and to better describe the S-type cell phenotype [21]. This study included the SK-N-SH cell line and the LAN-1 cell line, the only 2 out of the 17 studied cell lines that displayed 2 sub-populations of N- and S-type cells. N-type cells presented activity for enolase and expressed chromogranin, MC25, and AO10 antigens and the NGF receptor, whereas S-type cells did not. Nine antigens were found to be strongly expressed and specific to the S-type, as for example mel-CSPG, β2-microglobulin, and HLA class I antigens. The expression pattern of cell surface antigens of S-type cells was compared with that of various normal or tumor cells of neuroectodermal or mesenchymal origin. Interestingly, S-type cells most closely resembled the phenotype of cultured meningeal cells that are ectomesenchymal cells deriving from neural crest cells. In addition, interconversion between the N- and S-types was associated with coordinated changes in expression of the analyzed cell surface markers as well as changes in biochemical activity. Altogether, these data therefore suggested that the interconversion between an N-type and an S-type cell is associated with two specific differentiation programs rather than being the result of a random phenotypic instability of cultured cells [21].

Of note, it may be highlighted that the definition of the S-type was not as clear as that of the N-type, with studies referring either to melanocytic, Schwannian, meningeal, glial [17], neuronal, or mesenchymal properties of these cells [16,18,21]. Moreover, whereas N- and S-type cells have been observed in various other morphologically heterogeneous neuroblastoma cell lines, a subset of sub-lines of S-type could only grow for a limited number of passages and did not give rise to immortal neuroblastoma cell lines [15]. This suggested that some S-type cell lines may operate terminal differentiation; yet such a mechanism has not been fully explored.

### 2.3. Intermediate I-Type Cells

A third cell state, called I-type for “intermediate” type has been further described in several neuroblastoma cell lines, such as the SK-N-BE(2)-C cell line [15]. I-type cells are morphologically and biochemically intermediate between S-type and N-type cells. These cells are moderately adherent with relatively scant cytoplasm and variable neuritic processes, and they express vimentin and some noradrenergic enzymes [22]. It has been suggested that I-type cells may represent a cellular intermediate in the N/S interconversion [15]. Alternatively, the I-type could represent a stem cell from which both N- and S-type cells may arise. Interestingly I-type cells have been shown to represent the most aggressive subpopulation within a tumor [22,23], and they show properties of stem-like cells, including the expression of stem cell markers such as CD133. High-risk neuroblastoma tumors had higher levels of expression of cells with I-type features than tumors from low-risk cases. The I-type population may represent an important intermediate cell state potentially involved in transdifferentiation and subsequent neuroblastoma heterogeneity (for a recent review, see [24]).

## 3. The Various Phenotypes of Neuroblastoma Cells Are Associated with Specific Transcriptomic and Epigenetic States: Characterization of Core Regulatory Circuitries (CRC) in Neuroblastoma

### 3.1. Epigenetic and Transcriptomic Profiles Define Noradrenergic (NOR) and Mesenchymal (MES) Identities

More than 40 years after the first description of the two morphological N-type and S-type phenotypes, in 2017, two teams provided major advances in the characterization of the molecular features of these two states [19,25]. Both teams explored the transcriptomic and epigenetic landscapes of a panel of neuroblastoma cell lines and also studied human neural crest cell (hNCC) lines. ChIP-seq analysis of the H3K27 acetylation mark allowed the identification of super-enhancers (SEs) consisting of dense clusters of enhancers that have been shown to underlie cell and lineage identity [26]. Relying on the SE profiles, both studies demonstrated that neuroblastoma cells may adopt two distinct identities consistent with the previous phenotypic observations: a neuronal sympathetic identity associated with SEs and expression of adrenergic/noradrenergic markers such as PHOX2B, TH, and DBH or an undifferentiated identity, closely resembling NCC lines, associated with SEs and expression of mesenchymal markers such as vimentin or fibronectin (Figure 1C). Boeva and colleagues called this identity NCC-like, whereas van Groningen and colleagues referred to it as mesenchymal. We will further use the term mesenchymal (MES) to name the second identity since it refers to a more general feature in the field of oncology than the more specialized NCC-like term. We will use the term noradrenergic (NOR) and not adrenergic to refer to the sympathetic identity with noradrenergic properties. Indeed, adrenergic cells are characterized by the expression of the PNMT (phenylethanolamine N-methyltransferase) enzyme that converts noradrenaline to adrenaline. This enzyme is mostly restricted to chromaffin cells and is not expressed by NOR sympathetic neurons. Of note, the *PNMT* gene is not or weakly expressed at the mRNA level in the series of neuroblastoma cell lines studied by Boeva and colleagues [19], and another study showed that PNMT was not present in any of the analyzed neuroblastoma cell lines (*n* = 25), as evaluated by Western blot [27].

The epigenetic and transcriptomic landscapes of the SH-SY5Y and SH-EP cell lines were classified by both teams as NOR and MES, respectively, confirming the seminal description of these cell lines. Boeva and colleagues mostly characterized cell lines established in culture for a long time in the presence of serum, whereas van Groningen and colleagues used the CD133 marker to characterize new isogenic pairs of NOR and MES cell lines from primary tumors or invaded bone marrow of neuroblastoma patients, cultured under serum-free conditions [25,28]. Interestingly, the parental heterogeneous SK-N-SH cell line appeared in an intermediate group of cell lines between the NOR/group I and MES/group II (Figure 1C) [19]. Single-cell RNA-sequencing (scRNA-seq) analyses confirmed that the SK-N-SH cell line contained both cell types, which explained the intermediate pattern of SEs [29,30]. On the contrary, the SK-N-BE(2)-C cell line originally described as an intermediate I-type cell line clustered within the noradrenergic group [19]. Whether cell lines described as I-type or intermediate express a strong NOR signature and co-express low levels of mesenchymal markers or include two or more different populations remains to be investigated.

### 3.2. The Core Regulatory Circuitry of Noradrenergic Cells Includes Major Transcription Factors Involved in the Development of the Sympathetic Nervous System

**Figure 2:**
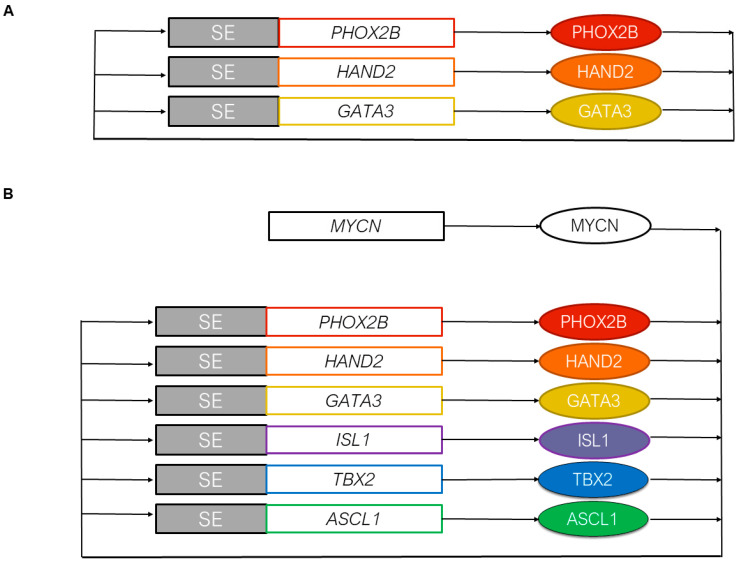
Core regulatory circuitries of transcription factors defining the noradrenergic identity. (**A**) The initial described CRC includes PHOX2B, HAND2, and GATA3 that form a positive feedback, interconnected co-regulatory loop [19]. (**B**) In the *MYCN*-amplified SK-N-BE(2)-C neuroblastoma cell line, ISL1, TBX2, and ASCL1 have been added to the initial CRC [32,33,34]. MYCN regulates each of these genes, yet it remains tricky to determine whether *MYCN* itself is regulated by an SE due to its high copy number level in *MYCN*-amplified cell lines. SEs and gene loci are represented by rectangles, and proteins are represented by oval symbols.

CRCs associated with both identities could then be defined from the SE landscapes. CRCs are TF networks with feed-forward auto-regulatory loops that establish cell fate decisions and maintain cell identity [26]. The CRC governing the sympathetic noradrenergic identity is now well established with consistent results from several studies. Analysis of the SEs characterized in the NOR identity first suggested that the NOR CRC contained the PHOX2A/PHOX2B, HAND1/HAND2, TBX2, ISL1, and GATA3 TFs, among others [19,25]. Importantly, PHOX2B, HAND2, and GATA3 are known to be key TFs involved in the specification and differentiation of the noradrenergic sympathetic nervous system [31]. ChIP-seq for the PHOX2B, HAND2, and GATA3 TFs in the neuroblastoma CLB-GA cell line confirmed that binding regions for these three TFs corresponded to the H3K27ac peaks and SEs of the NOR identity. PHOX2B, HAND2, and GATA3 are SE-regulated and bind to one another’s SE, defining a specific NOR CRC module (Figure 2A) [19]. This module has been confirmed with HAND2, ISL1, PHOX2B, GATA3, and TBX2 and has been shown to form a positive feedback, interconnected co-regulatory loop in the *MYCN*-amplified SK-N-BE(2)-C neuroblastoma cell line (Figure 2B) [32]. This study suggested that MYCN regulates each of these genes, yet it remains tricky to determine whether MYCN itself is regulated by an SE due to its high copy number level in *MYCN*-amplified cell lines. Importantly, the aforementioned TFs were part of a large list of candidate genes for which selective dependencies have been revealed in *MYCN*-amplified neuroblastoma cell lines using an unbiased genome-scale CRISPR-Cas9 screen [32]. These TFs therefore appear to be important both for cell state and survival.

As mentioned above, TBX2 (T-box 2 transcription factor) is a constituent of the NOR CRC in neuroblastoma cells, together with HAND2, GATA3, and PHOX2B (Figure 2B) [32,33]. Although not previously reported to play a role in neuroblastoma oncogenesis, *TBX2* is a well-known developmental gene acting in various tissues, including neural crest [35]. Of note, TBX2 is encoded by a gene residing on chromosome 17q for which highly recurrent gains have been demonstrated in high-risk human neuroblastoma [36]. This suggested that TBX2 is a dosage-sensitive CRC TF that may confer a selective advantage to tumors cells exhibiting 17q gain. Indeed, in high-stage diseases, significantly higher *TBX2* expression levels are due to increased *TBX2* copy number [33]. Further experiments documented that both MYCN and TBX2 are able to increase FOXM1 expression and activity, promoting the switch of DREAM (dimerization partner, RB-like, E2F, and multi-vulval class B) complex to an active form driving cell cycle progression. Finally, a combination of THZ1, a covalent inhibitor of CDK7 [37], with JQ1, a bromo-domain inhibitor [38], induced a strong synergistic effect on cell growth and apoptosis in several neuroblastoma cell lines. A strong collapse of the whole CRC and loss of FOXM1 repression of *E2F/DREAM* complex genes were documented following this treatment [33]. The same combination was shown to disable the CRC of SK-N-BE(2)-C cells both in vitro and in vivo, with rapid downregulation of the associated TF gene expression [32].

*ASCL1* has been identified as a NOR SE-associated TF gene [25]. This gene encodes a basic Helix-Loop-Helix (bHLH) TF, known to be expressed in sympathetic progenitors and silenced when cells differentiate into neurons or chromaffin cells during normal development of the peripheral nervous system [39,40]. Recently, ASCL1 has been shown to be a new member of the NOR CRC (Figure 2B) [34]. Indeed, ASCL1 binds to the associated SEs and regulates the expression of the other members of the NOR CRC (HAND2, ISL1, PHOX2B, GATA3, and TBX2), in concert with MYCN and LMO1. As with many other members of this CRC, *ASCL1* has previously been identified as a selective neuroblastoma dependency gene [32].

### 3.3. MYCN and the Epigenetic Landscape of Neuroblastoma Cells

*MYCN* amplification is frequently observed in neuroblastoma primary tumors and cell lines, and *MYCN* over-expression has been shown to drive tumorigenesis in a transgenic mouse model when placed under the control of the TH promoter [41]. The link between *MYCN* expression and the N- and S-phenotypes was first reported in the NBL-W cell line. This cell line derived from a primary tumor of a patient with a stage 4S metastatic neuroblastoma has been described to also contain two morphologically distinct N- and S-type populations [42]. Interestingly, all cells of the NBL-W cell line presented with an amplification at the *MYCN* locus with around 100 copies of the *MYCN* gene at the 2p15 location. Nonetheless, high expression levels of MYCN mRNA and protein were only seen in N-type cells, and levels were decreased in S-type cells. Thus, the sub-clones were of identical genotypes, but there was a differential expression of MYCN between the N- and S-types. This suggested that spontaneous downregulation of MYCN expression can occur in a cell with a high amplification of the oncogene. In the SK-N-SH cell line, despite the absence of *MYCN* amplification, the MYCN mRNA and protein were present in N-type cells, whereas lower levels were detected in the S-type cells [43]. Altogether, these data suggested that the differential MYCN expression could be involved in the cell interconversion between both phenotypes, although no functional data have been shown to support such a hypothesis.

In the analysis of Boeva and colleagues of the 18 cell lines of NOR identity, *MYCN* SEs were identified in 10 samples, with or without amplification and no specific group could be linked to the *MYCN* status [19]. A recent study analyzed the consequences of MYCN over-expression or withdrawal in neuroblastoma cells at the genome-wide level using the well-characterized tet-off MYCN SHEP-21N cell line model [44] and, additionally, a tet-on MYCN model in the parental SH-EP neuroblastoma cell line [45]. ChIP-seq for MYCN was also performed across several *MYCN*-amplified neuroblastoma lines to generate a consensus map of around 10,000 regions that exhibited strong MYCN occupancy. Physiologically expressed, i.e., when not in excess, MYCN binds to consensus strong canonical CACGTG E-boxes at promoters promoting growth and proliferation. When expressed in excess (which is the case when the gene is amplified), it also binds and invades weaker CANNTG E-boxes. Distal enhancers are therefore a reservoir for MYCN binding when its expression is deregulated. Kinetic analysis demonstrated that MYCN induction established the promoter gene signatures before the enhancer gene signatures. *MYCN* amplification in neuroblastoma can therefore induce a dose-dependent increase in the expression of genes with weak MYCN binding sites in their enhancers, thus promoting tumor-specific expression of MYCN target genes [45].

Another study, based on the transcriptomic profiles of high-risk neuroblastoma and considering clinical and biological features, characterized three molecular subtypes of neuroblastoma: a first subtype mostly presenting with *MYCN* amplification, a second type with hemizygous deletions of chromosome 11q, and a third type not strongly associated with specific genomic alterations but displaying a strong mesenchymal signature [46]. The predominantly MYCN-amplified subtype corresponded to phenotypically undifferentiated cells with a high mitosis-karyorrhexis index (MKI). Further experiments identified a TEAD4-MYCN positive feedback loop as a master driver of this *MYCN*-amplified subtype [46]. Nevertheless, TEAD4 activity in this context seemed to be independent of its well-characterized involvement in the HIPPO pathway through YAP/TAZ activation. Interestingly, TEAD4 was shown to regulate MYCN in a positive way, not only at the transcriptional level but also at a posttranslational level. Finally, silencing of either MYCN or TEAD4 largely modified the transcriptional signature of the cells toward a neuroblastoma stage 1 signature with decreased viability in vitro and in vivo.

### 3.4. Transcription Factors Associated with the Mesenchymal Identity and Chemoresistance of Mesenchymal Cells

The MES identity is less well characterized in terms of CRC. This may be due to the smaller number of mesenchymal neuroblastoma cell lines available in the scientific community and included in the aforementioned studies. Indeed, the list of TFs expected to participate in the CRC module of MES cells is only partially overlapping between the studies of Boeva and colleagues and van Groningen and colleagues, and no ChIP-seq data for specific TFs of the MES identity have been published up to now. On the one hand, AP-1 family members, IRF1/IRF2/IRF3 and RUNX1/RUNX2 TFs, among others, were identified as CRC members in the MES cell lines in one study [19], whereas on the other hand, MEOX1/2, SIX1/SIX4, SOX9, SMAD3, and WWTR1, among others, were highlighted in the second study [25]. Interestingly the PRRX1 TF was identified as a MES-related identity TF in both papers. PRRX1 is an homeobox protein that has been shown to induce EMT in breast cancers [47]. Over-expression of PRRX1 in NOR cells has been shown to modify cell identity toward a more MES state [25], which is further discussed below.

Importantly, the characterization of the NOR and MES identity revealed that MES cells were more resistant in vitro to standard chemotherapeutic agents used to treat neuroblastoma patients, such as cisplatin, doxorubicin, and etoposide [19,25]. This was documented by comparing the sensitivity of isogenic cell lines of NOR and MES identity to various agents. In addition, an enrichment of MES cells was documented in the heterogeneous SK-N-SH cell line after in vitro treatment [19]. An enrichment of PRRX1 positive cells has been reported in a few post-chemotherapy and relapse biopsies from patients [25]. Nevertheless, the demonstration that these cells are tumor cells exhibiting genetic alterations and not normal mesenchymal cells remains to be obtained. These data suggest that therapy may exert selective pressure and that resistant MES cells could escape actual chemotherapies. However, it cannot be excluded that cells of NOR identity could transdifferentiate toward a MES identity upon treatment in patient tumors. Since tumors at relapse are not systematically enriched in MES cells [19], plasticity may also be involved in the reversion of cell identity after treatment, MES cells switching back to a NOR state. Spontaneous and induced plasticity of neuroblastoma cells is further discussed below.

## 4. Plasticity from a Noradrenergic to a Mesenchymal Cell Identity in Neuroblastoma Defines a Noradrenergic-to-Mesenchymal Transition (NMT)

### 4.1. Spontaneous Plasticity

Plasticity between the NOR and MES states associated with different epigenetic landscapes clearly corresponds to the previously described transdifferentiation or interconversion between the N- and S-types. The aforementioned experiments indeed suggested a bi-directional and spontaneous ability of cells obtained from the emblematic SK-N-SH model to transdifferentiate. Very recently, single-cell RNA-sequencing analysis of the SK-N-SH cell line further characterized the two cell populations and identified CD44 as a specific cell surface marker of the MES identity [29]. Both sorted NOR/CD44^neg^ and MES/CD44^pos^ populations were able to give rise to heterogeneous cell cultures, confirming the spontaneous and bidirectional plasticity of these cells. 

Plasticity between NOR and MES state is not a property exclusive to the SK-N-SH model. Recent results reported spontaneous plasticity in several other models between both identities and showed that plasticity relies on epigenetic reprogramming. An interconversion mechanism was reported for the heterogeneous cell line AMC700B using FACS sort with the CD133 marker [25]. Single CD133^neg^/NOR sorted cells were able to expand and formed several clones comprising up to 34% of CD133^pos^/MES cells. Interestingly, single sorted CD133^pos^ cells were not able to expand and form clones; yet, sorted and batch-cultured CD133^pos^ cells reconstituted a heterogeneous population including 51% of CD133^neg^ cells after eight passages. Both CD133^pos^ and CD133^neg^ sorted cells were able to generate heterogeneous tumors, comprising NOR cells expressing DBH and MES cells expressing vimentin when subcutaneously injected in mice. 

**Figure 3:**
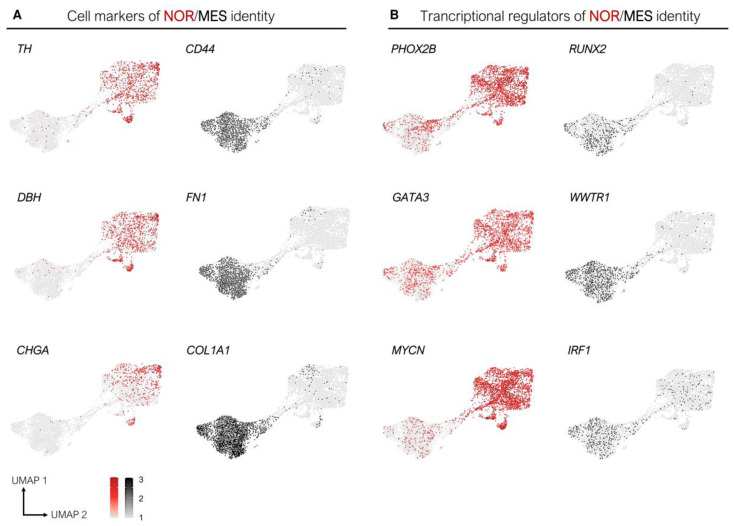
The IC-pPDXC-63 cell line is biphenotypic in vitro and resumes expression of known specific cellular markers and transcription regulators of the NOR and MES identities. UMAP representation of the single-cell RNA-sequencing of the IC-pPDXC-63 model, with each dot corresponding to a cell [29]. (**A**) The *TH* and *DBH* enzymes as well as the neuroendocrine secretory protein *CHGA* are expressed in noradrenergic cells, while *CD44, COL1A1*, and *FN1* are specifically expressed in cells of mesenchymal identity. (**B**) The transcriptional regulators *PHOX2B*, *GATA3*, and *MYCN* are specifically expressed in noradrenergic cells, while *RUNX2*, *WWTR1*, and *IRF1* exhibit the opposite expression pattern.

New cell lines have also been generated from PDX models and will be useful for studying molecular mechanisms involved in the plasticity of neuroblastoma cells. In particular, the IC-pPDXC-63 cell line, derived from the noradrenergic IC-pPDX-63 model, was biphenotypic in culture, with floating neurospheres expressing PHOX2B and adherent cells expressing CD44 [29]. Interestingly, this cell line could resume the observations of pioneer papers with differential expression of cellular markers, such as TH and DBH in the neurospheres, while collagenases and fibronectin were expressed specifically in the adherent cells (Figure 3A). Expression of some known transcription factors for both identities are also consistent with the recent observations [19,25]. This *MYCN*-amplified model confirmed the specific *MYCN* downregulation in the mesenchymal cluster of cells (Figure 3B). These results revealed the plasticity potential of a sub-population of NOR tumor cells able to give rise to MES cells in culture. Conversely, our recent results demonstrate that the in vivo microenvironment provides a strong pressure toward the NOR identity, as cells of MES identity from different models transdifferentiate into a NOR identity when xenografted in mouse [29]. Consistent with these observations, tumor cells of mesenchymal identity were not detected in PDX models.

Altogether these data documented the plasticity of a subset of neuroblastoma cells that are able to switch spontaneously from an identity to another in vitro and in vivo, relying on specific transcriptomic and epigenetic programs.

### 4.2. Induction of a Mesenchymal State in Neuroblastoma Cells In Vitro by Drug Treatments

In addition to spontaneous plasticity between the NOR and MES states, a few studies have explored drug resistance in neuroblastoma cell lines in vitro and described changes considered as an EMT following treatment of cells with chemotherapy agents or targeted therapies.

EMT, first described in 1982 [48], is an important mechanism during metazoan embryogenesis [49], consisting of multiple events by which epithelial cells lose many of their proper characteristics to become mesenchymal cells characterized by increased resistance to apoptosis, reduced cell–cell adhesion, and activation of proteolysis and motility. Aberrant reactivation of EMT has been deeply investigated during oncogenesis and has been shown to fuel tumor initiation and promote invasion and metastasis during tumor development in many cancer types [50]. In addition, tumor cells with mesenchymal features have been shown to exhibit increased resistance to chemotherapy [51]. EMT is induced by various molecular, cellular, and microenvironmental signals. Commonly used molecular markers for EMT include increased expression of N-cadherin and vimentin, phosphorylation of β-catenin that translocates into the nucleus activating EMT target genes, and increased production of TFs such as SNAIL (*SNAI1*), SLUG (*SNAI2*), TWIST1, TWIST2, ZEB1, and ZEB2 [52]. Loss of E-cadherin expression in epithelial cells is considered a key event in EMT [53]. 

In neuroblastoma, cisplatin resistant sub-lines of SK-N-AS, Kelly, and CHP-212 neuroblastoma cell lines have been generated by exposing cells to increasing doses of cisplatin for several months [54]. Cisplatin-resistant cell lines exhibited changes in morphology, modifications in the abundance of cytoskeletal proteins, and increased in vitro invasion, such changes being consistent with those associated with EMT. Of note, cisplatin induced cross resistance to temozolomide, etoposide, and irinotecan [54]. In another study, resistant clones of the noradrenergic SH-SY5Y cell line bearing an ALK F1174L-activating mutation were obtained after long treatment with increasing doses of two ALK inhibitors, TAE684 and LDK378 (ceritinib) [55]. Over-expression and activation of the AXL tyrosine kinase and acquisition of an EMT phenotype were documented in the resistant cells. Indeed, resistant cells were elongated, spindle shaped, and exhibited decreased cell-to-cell contact. Moreover, they over-expressed the key EMT inducers TWIST2 and SNAI2 and the typical MES markers vimentin and fibronectin. Further inhibition of AXL led to decreased growth and invasiveness of the resistant cells with a concomitant decrease in ERK/MAPK signaling. Thus, AXL over-expression was associated with activation of the ERK/MAPK signaling pathway and development of EMT. Interestingly, ectopic expression of AXL or TWIST2 in the TAE684-sensitive SH-SY5Y parental cells resulted in elevated expression of mesenchymal markers and invasive capacity.

**Figure 4:**
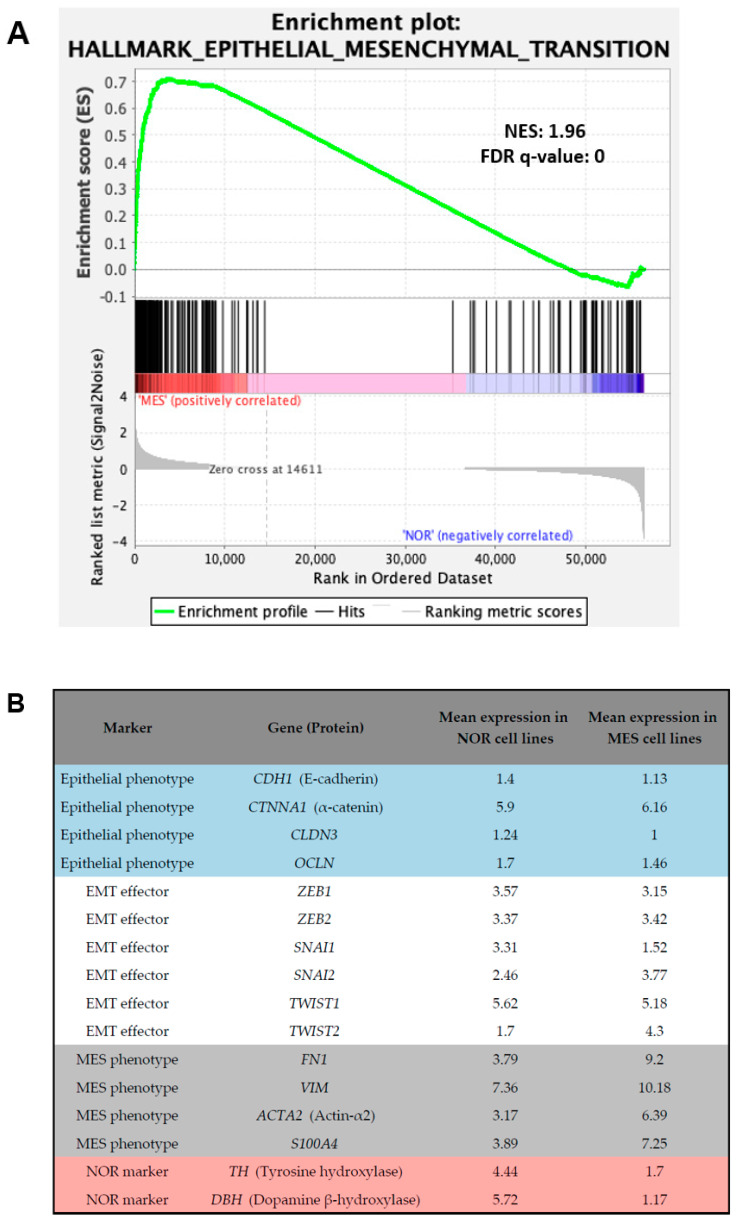
Neuroblastoma MES cells express typical mesenchymal markers but not EMT effectors. (**A**) GSEA analysis on noradrenergic and mesenchymal neuroblastoma cell lines revealed the hallmark category “epithelial-to-mesenchymal transition”. (**B**) Mean of the normalized expression values (Log2-transformed (FPKM + 2)) obtained from RNA-seq data in the same cell lines for epithelial markers, EMT effectors, and MES and NOR markers. Data are from [19].

The acquisition of mesenchymal markers and migratory and invasive properties by neuroblastoma cells after various drug treatments has been considered as EMT. Nonetheless, neuroblastoma is a neuroendocrine cancer and cannot be considered as a carcinoma developing from epithelial cells. Interestingly, a gene set enrichment analysis (GSEA) performed on NOR and MES neuroblastoma cell lines using our previously published dataset [19] highlighted the hallmark “epithelial-to-mesenchymal transition” as enriched in MES cell lines (Figure 4A). Genes present in the core enrichment of this set of genes in samples with MES identity included the typical MES markers fibronectin and vimentin, several collagens, and integrins (Figure 4B and Table S1). Most of the emblematic markers of an epithelial phenotype, such as E-cadherin and tight-junction molecules including claudins and occludin are not expressed in neuroblastoma cells. Among the known EMT effectors, only *TWIST2* exhibited an increased expression in MES cell lines compared with NOR cell lines (Figure 4B and Table S1). The term EMT is therefore not fully relevant for describing the induction of a MES state in neuroblastoma.

### 4.3. Genetically Induced Plasticity: From a Noradrenergic Identity to a Mesenchymal Identity

Recent studies have considered the NOR and MES identities of neuroblastoma cells and further described and investigated plasticity between both states. Genetically induced plasticity from a NOR state to a MES state has been reported following either over-expression of the PRRX1 TF or NOTCH3-IC (intracellular domain) [25,56]. Over-expression of the PRRX1 TF in SK-N-BE(2)-C cells reprogrammed their SE landscape toward a MES identity [25]. Morphological changes and expression of the EMT marker SNAI2 were observed upon PRRX1 over-expression. Conversely, repression of NOR markers such as PHOX2B and DBH was observed in these cells. Nevertheless, the silencing of PRRX1 in MES cells did not induce a transdifferentiation toward a NOR identity, suggesting that depletion of PRRX1 alone in MES cells is not sufficient to induce a switch. Other master regulators of the MES identity may be important in the maintenance of this state and may also have to be depleted to induce plasticity toward the NOR identity. Another hypothesis is that depletion of the PRRX1 MES master regulator would not be sufficient and has to be accompanied by over-expression of one or several NOR master regulators. Recently, reprogramming of noradrenergic cells into mesenchymal cells has been performed by expressing NOTCH3-IC in SH-SY5Y cells. NOTCH pathway genes indeed appeared co-regulated within the MES state in neuroblastoma cell lines. Moreover, reprogramming was linked to downregulation of the expression of noradrenergic CRC TFs with severe reduction of H3K27ac at their SEs. Consistently, in SH-SY5Y reprogrammed cells, new enhancers were acquired at similar positions as in mesenchymal SH-EP cells [56]. However, the consequences of a *NOTCH3* knock-out in MES cells have not been explored. These results highlight that in vitro over-expression of a MES factor induces an epigenetic reprogramming from a NOR toward a MES state, whereas downregulation of a MES factor in MES cells seems insufficient for inducing a NOR identity. 

By using a CRISPR-Cas9 approach, genetic knock-out of TFs of the NOR CRC were recently performed in the SH-SY5Y cell line. While knock-out of *PHOX2A* or *PHOX2B* did not result in changes in the phenotype of the cells, *GATA3* knock-out induced a shift toward a mesenchymal phenotype characterized by an abundant cytoplasm and many actin stress fibers [29]. These GATA3^−^/^−^ cells showed a consistent increase in invasion and migration as measured by 3D spheroid assay and transwell assay, respectively, and higher resistance to chemotherapy compared with the parental SH-SY5Y cell line. Importantly, GATA3^−^/^−^ clones showed a strong reduction in the expression of the TFs of the NOR CRC, including PHOX2A, PHOX2B, and HAND2 [29], and bulk RNA-seq analysis showed that these cells exhibited a mesenchymal transcriptomic profile, as they were close to the SH-EP cell line. Consistently, these cells presented with an SE landscape similar to the one of MES neuroblastoma cell lines. These results therefore demonstrate plasticity of the noradrenergic SH-SY5Y cell line that is able to transdifferentiate toward a mesenchymal identity upon *GATA3* knock-out. 

ARID1A, for AT-rich interacting domain-containing protein 1A, is a subunit of the SWI/SNF chromatin remodeling complex. In 2013, chromosomal deletions and sequence alterations of the chromatin-remodeling genes *ARID1A* and *ARID1B* were reported in 11% of neuroblastoma cases and were shown to be associated with decreased survival [57]. More recently, *Mycn* over-expression has been shown to select for loss of Arid1a during tumorigenesis in a model of primary mouse NCCs [58]. Interestingly, in the *MYCN*-amplified NGP neuroblastoma cell line, depletion of *ARID1A* using CRISPR-Cas9 promotes the transdifferentiation of NOR cells toward a MES state with increased invasion, migration, and resistance to cisplatin [59]. Consistently, ChIP-seq data for the H3K27ac mark confirmed the transdifferentiation with changes in the enhancer-mediated gene expression. In particular, reduced and increased levels of H3K27ac mark were observed at the *PHOX2B* and *FN1* loci, respectively. mRNAs and protein levels of PHOX2B were decreased, while those for fibronectin were increased in *ARID1A* knock-out cells [59].

## 5. Conclusion: Noradrenergic-to-Mesenchymal Transition (NMT) Rather Than Epithelial-to-Mesenchymal Transition (EMT) in Neuroblastoma

**Figure 5:**
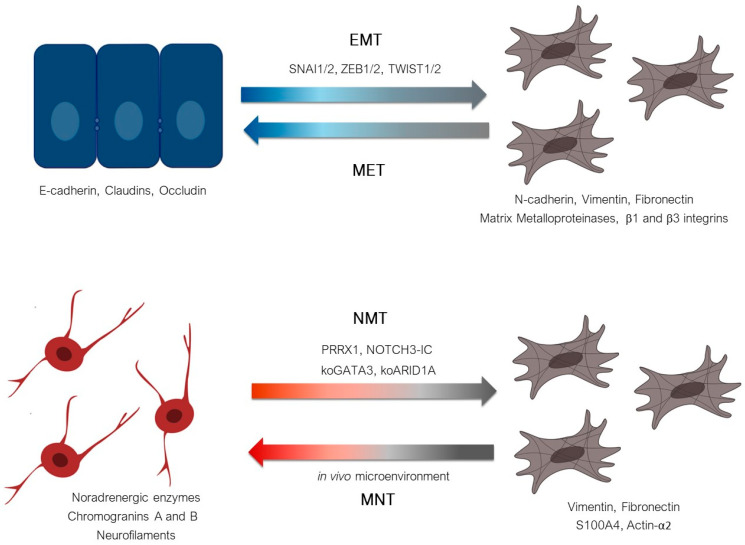
Plasticity from a noradrenergic to a mesenchymal identity in neuroblastoma defines a NOR-to-MES transition (NMT) reminiscent of the epithelial-to-mesenchymal transition (EMT). EMT, controlled by EMT effectors (SNAI1/2, ZEB1/2, TWIST1/2), refers to a shift from an epithelial cell expressing the emblematic marker E-cadherin to a cell expressing various mesenchymal markers. MET is known to be the reversion of EMT. NMT refers to the mechanism by which NOR cells transdifferentiate toward MES cells. Neuroblastoma NOR cells express typical neurofilament proteins and chromogranins as well as TH and DBH. MES neuroblastoma cells exhibit expression of typical mesenchymal markers such as vimentin and fibronectin. PRRX1 or NOTCH3-IC over-expression, as well as knock-out of *GATA3* or *ARID1A*, have been shown to be able to induce NMT. The reverse mechanism is thus called MNT.

Altogether, these data suggest that plasticity from the NOR to the MES cell identity in neuroblastoma defines a noradrenergic-to-mesenchymal transition (NMT) reminiscent of but different from EMT (Figure 5). Data obtained in our laboratory also indicate that neuroblastoma cells could transdifferentiate from a mesenchymal identity toward a noradrenergic one, thus defining a mesenchymal-to-noradrenergic transition (MNT), again reminiscent of but different from the mesenchymal-to-epithelial transition (MET) known to be the reversion of EMT [60] (Figure 5). In vitro, several studies documented the acquisition of MES properties by neuroblastoma cells with acquired resistance to standard chemotherapeutic agents or to targeted therapy against the ALK receptor. Using IHC with PRRX1 staining, van Groningen and colleagues reported that MES cells can be enriched in tumors after treatment [25]. Nonetheless, whether these cells are tumor cells bearing genetic alterations remains to be fully demonstrated. Since MES cells do not express the typical markers (PHOX2B, TH, or DBH) used in clinical routine to characterize neuroblastoma cells, their detection requires the use of other markers or technics. To identify such MES cells potentially involved in minimal residual disease, one study recently proposed the use of a panel including *PRRX1*, *POSTN*, and *FMO3* and documented their mRNA expression in peripheral blood, bone marrow, and peripheral blood stem cell samples of neuroblastoma patients using RT-qPCR [61]. Strikingly, the MES markers were more prevalent in the bone marrow of neuroblastoma patients who ultimately experienced relapse, but the MES markers were rarely present at the actual time of relapse. These interesting results require confirmation since the used markers are not fully specific for mesenchymal tumor cells. 

Single-cell transcriptomics represents a powerful tool for dissecting tumor heterogeneity and is currently used in the field. Several recent analyses of neuroblastoma cases provided a picture of the transcriptome of tumor cells at the single-cell level [30,62,63,64,65,66]. No cluster of MES tumor cells as defined in vitro could be detected in three cohorts of 17, 16, and 21 neuroblastoma cases from all stages [30,62,63]. Consistent with these observations, our recent data document a strong pressure of the environment toward a NOR identity in vivo in various models, including PDXs [29]. Nevertheless, we cannot exclude that a biological and/or technical bias of tumor sampling in terms of technics, spatial localization, or temporal variability after treatments may explain the absence of MES tumor cells. Another hypothesis relies on such cells presenting with a subtle signature of plasticity potential that remains to be explored. Interestingly, those studies also addressed the question of the cell of origin of neuroblastoma by dissecting the human developing adrenal medulla, as around half of these tumors arise in the adrenal gland. Despite confusion in cell labelling by Dong et al. [62,67,68,69,70], several studies converge on the idea that tumor cells resemble normal fetal adrenal neuroblasts [30,62,63]. Of note, few cases contained NOR tumor cells with traits of MES cells [30], Schwann-cell precursors or bridge cells [64,65], or post-natal progenitor cells [66], suggesting either distinct cells of origin or a certain plasticity potential that remains to be understood. Deconvolution analyses of bulk RNA-seq data using human developmental signatures revealed that high-risk and low-risk neuroblastomas are mostly composed of immature or more differentiated neuroblast-like tumor cells, respectively [30,62,64,71].

Altogether, the plasticity observed in vitro and the heterogeneity of tumor cells in patients suggest that some NOR tumor cells have a potential of differentiation in more migratory and chemoresistant MES cells, which could be involved in metastasis or relapse after treatments. Ongoing analyses focusing on neuroblastoma models of plasticity [29] will allow the molecular and cellular mechanisms of reprogramming between NOR, MES, and intermediate identities to be deciphered. In parallel, the use of single-cell approaches in close collaboration with clinicians will allow cell identity to be caught at different time points during disease evolution, before and after treatments, and on distinct localizations whenever possible with a very low amount of material. The integration of mechanistic studies and single-cell characterization of patient tumors will undoubtedly identify the key actors of plasticity along an NMT–MNT spectrum and potential new biomarkers and therapeutic targets. Pre-clinical studies using PDX models will validate the most promising candidates before prospective clinical trials.

## Supplementary Materials

The following are available online at https://www.mdpi.com/article/10.3390/cancers13122904/s1, Table S1. Mean of the normalized expression values (Log2-transformed (FPKM + 2)) obtained from RNA-seq data in 18 noradrenergic neuroblastoma cell lines and 3 mesenchymal neuroblastoma cell lines for epithelial markers, EMT and NMT effectors, mesenchymal and noradrenergic markers. Data are from Boeva et al. [19].
