# Supplementary material for: Plasticity in Neuroblastoma Cell Identity Defines a Noradrenergic-to-Mesenchymal Transition (NMT)
Source: Cancers (Basel). 2021 Jun 10;13(12):2904. doi: 10.3390/cancers13122904 (PMC8230375; doi:10.3390/cancers13122904)
Supplement: Supplementary file 1 [file cancers-13-02904-s001.zip › cancers-1230770-supplementary.pdf]

Supplementary Material: Plasticity in Neuroblastoma Cell Identity Defines a Noradrenergic-to-Mesenchymal Transition (NMT)

Margot Gautier, Cécile Thirant, Olivier Delattre and Isabelle Janoueix-Lerosey

Table S1. Mean of the normalized expression values (Log2-transformed (FPKM+2)) obtained from RNA-seq data in 18 noradrenergic neuroblastoma cell lines and 3 mesenchymal neuroblastoma cell lines for epithelial markers, EMT and NMT effectors, mesenchymal and noradrenergic markers. Data are from "Heterogeneity of neuroblastoma cell identity defined by transcriptional circuitries" by Boeva, V. et al., 2017, Nature Genetics, 49, 1408-1413, Copyright [2021] by Springer Nature [19].

Marker	Gene (Protein)	Mean expression in NOR cell lines	Mean expression in MES cell lines
Epithelial phenotype	*CDH1* (E-cadherin)	1.4	1.13
Epithelial phenotype	*CTNNA1* (α-catenin)	5.9	6.16
Epithelial phenotype	*JUP* (Ɣ-catenin)	5.21	2.34
Epithelial phenotype	*CLDN1* (Claudin 1)	1.3	2.9
Epithelial phenotype	*CLDN3* (Claudin 3)	1.24	1
Epithelial phenotype	*CLDN4* (Claudin 4)	1.52	2.32
Epithelial phenotype	*CLDN7* (Claudin 7)	1.28	1.99
Epithelial phenotype	*OCLN* (Occludin)	1.7	1.46
EMT effector	*ZEB1*	3.57	3.15
EMT effector	*ZEB2*	3.37	3.42
EMT effector	*SNAI1*	3.31	1.52
EMT effector	*SNAI2*	2.46	3.77
EMT effector	*TWIST1*	5.62	5.18
EMT effector	*TWIST2*	1.7	4.3
MES phenotype	*FN1* (Fibronectin)	3.79	9.2
MES phenotype	*VIM* (Vimentin)	7.36	10.18
MES phenotype	*ITGB1* (Integrin β1)	6.1	7.6
MES phenotype	*ITGB3* (Integrin β3)	1.59	1.82
MES phenotype	*ACTA2* (Actin-α2)	3.17	6.39
MES phenotype	*COL1A1*	2.73	7.76
MES phenotype	*COL1A2*	1.18	3.8
MES phenotype	*CDH2* (N-cadherin)	5.8	5.26
MES phenotype	*S100A4*	3.89	7.25
MES phenotype	*IRF1*	2.12	4.06
NMT effector	*PRRX1*	1.2	3.86
NOR marker	*PHOX2B*	7.15	1.1
NOR marker	*HAND2*	8.2	5.1
NOR marker	*GATA3*	6.32	4
NOR marker	*TH* (Tyrosine hydroxylase)	4.44	1.7
NOR marker	*DBH* (Dopamine β-hydroxylase)	5.72	1.17
NOR marker	*CHGA*	6	1.14
NOR marker	*CHGB*	6.27	1.95
NOR marker	*NEFL*	4.78	1.15
NOR marker	*NEFM*	4.46	1.1
NOR marker	*NEFH*	3.8	1.2
